# Public attitudes towards sexual behavior–Results of the German Health and Sexuality Survey (GeSiD)

**DOI:** 10.1371/journal.pone.0282187

**Published:** 2023-03-17

**Authors:** Julia Ludwig, Franziska Brunner, Christian Wiessner, Peer Briken, Miriam G. Gerlich, Olaf von dem Knesebeck

**Affiliations:** 1 Center for Psychosocial Medicine, Institute of Medical Sociology, University Medical Center Hamburg-Eppendorf, Hamburg, Germany; 2 Center for Psychosocial Medicine, Institute for Sex Research, Sexual Medicine, and Forensic Psychiatry, University Medical Center Hamburg-Eppendorf, Hamburg, Germany; 3 Center for Experimental Medicine, Institute of Medical Biometry and Epidemiology, University Medical Center Hamburg-Eppendorf, Hamburg, Germany; 4 Federal Center for Health Education, Cologne, Germany; University of Technology Sydney, AUSTRALIA

## Abstract

Population-level data on predictors for attitudes towards sexual behavior are missing for Germany. The current study investigated sexual attitudes in the German population with regard to sociodemographic and sociocultural factors. Data originated from the German Health and Sexuality Survey (GeSiD; N = 4,955) carried out from October 2018 to September 2019. Computer-assisted face to face interviews were conducted with a large self-administered component due to the sensitive topics of the survey. Public acceptance towards several aspects of sexual behavior (extramarital sex, abortion, same sex sexual activities, sex work, promiscuity, sex without love) was investigated. Age, gender, education, religious aspects and migration background were introduced as predictors into logistic regression analyses. Overall, respondents rather rejected promiscuity (61%) and extramarital sex (81%) and rather supported same sex sexual activities (63–70%). Male respondents more likely rejected same sex sexual activities and abortion. Higher education was associated with more acceptance towards the sexual behaviors whereas first generation migrants, Muslim faith and religious devoutness were associated with less acceptance. Results indicate that gender is relevant in terms of sexual attitudes with male respondents tending to have more traditional and heteronormative gender role values. Furthermore, education, culture and religion play an important role concerning the liberality towards sexual behaviors. Acculturation processes of second generation migrants may lead to an adaptation of values. Comprehensive and culturally sensitive sex education may focus on differences concerning sexuality-related norms and values.

## Introduction

In the past decades, there has been a rapid shift of sexual attitudes associated with changing legislation in Germany [1]. For example, consensual same sex sexual interactions with a male adolescent under the age of 18 were illegal until 1994 [1]–therefore, the complete legal equalization of heterosexual and homosexual marriages was a significant step in 2017. Another recent milestone was the introduction of the third gender in Germany, leading to more equality for persons who cannot or do not want to assign themselves to either the male or the female sex [2]. Nonetheless, the legal basis is not the only indicator of acceptance towards sexual diversity and permissiveness [3]. Mechanisms behind sexual attitudes are shaped by different aspects, such as sociodemographic or cultural factors [4]. Up until now, sexuality, gender and reproduction seem to maintain the focus of controversial societal discourses. This is clearly illustrated by the ongoing debate about abortions which are illegal in Germany and only exempt from punishment under certain conditions (duty of consultation and abortion only up to 12 weeks after fertilization, medical and criminological indications) [5]. Ethical considerations influenced by the Protestant and Catholic church on the one hand, and the recognition of the pregnant woman´s decision as a reason for legitimacy on the other hand have been discussed controversially for five decades [6, 7]. Another example is the social and legal framework which maintained precarious working conditions for and cemented the stigmatization of sex workers for many centuries in Germany [8]. The introduction of the so called Prostitute Protection Act in 2017 helped decriminalize and protect sex workers [8]. However, it remains unclear how sex work is seen by the public and whether a changing legislation fosters liberal attitudes (for number of men who pay for sex in Germany see [9]).

In order to broaden the understanding of the mechanisms behind sexual attitudes, international studies have examined sociodemographic and cultural factors related to sexual attitudes in the past years [4, 10–14, 18].

They found increasing age to be associated with less permissive attitudes (e.g. concerning sex outside marriage, one-night stands or homosexuality) [4, 10]. Concerning acceptance towards lesbian, gay and bisexual persons in Germany, this age effect was also evident: Most accepting attitudes were expressed among those under 30 years and most negative attitudes among those over 60 years [11].

Results on gender differences concerning sexual attitudes are ambiguous: Study results from the U.S. overall found more permissive attitudes in male respondents, but differences in the response pattern occurred depending on the topic (e.g. casual sex was seen more acceptable among men but there were no gender differences in attitudes towards homosexuality) [12, 13]. Another large-scale study from the U.S. found similar results [4]. The overall acceptance towards sexuality-related topics was higher among male respondents, but acceptance towards same sex sexual actions was less pronounced among male respondents. The latter result is congruent with findings from the U.K. and Germany [10, 11, 14].

Little is known about the association of educational attainment and sexual attitudes. Initial evidence from the third wave of the National Survey of Sexual Attitudes and Lifestyles (NATSAL-3) conducted in Great Britain could demonstrate, that respondents with higher education reported more liberal views on non-exclusivity in marriage and homosexual sex compared to those with lower education, but towards one-night stands, the pattern was less clear [10].

Apart from the above mentioned sociodemographic factors, there is evidence on the relation of spirituality, religion, and sexual attitudes. Accordingly, spirituality and religiosity were found to be associated with more conservative sexual attitudes [15, 16]. However, recent study results were often limited to the U.S.-American context and mostly refer to young persons as they are most sexually active [15, 17, 18]. Therefore, conclusions about the association between religious devoutness and sexual attitudes cannot be made on an overall societal level.

Culture is another important aspect shaping attitudes and norms on sexuality-related topics [19]. Germany is a country of immigration and the German society is becoming more diverse [20], but it remains unclear how e.g. a history of migration may be linked to differences in sexual attitudes.

Therefore, the present study aims at assessing acceptance towards the topics “extramarital sex”, “consensual sex between two men/between two women”, “abortion”, “sex work”, “promiscuity” and “sex without love” in a population representative cross-sectional survey from Germany. Moreover, associations of gender, age, education, migration background, and religiousness with sexuality-related attitudes are drawn.

## Materials and methods

### Participants and data collection

Analyses were based on the first *German Health and Sexuality Survey* (GeSiD), which was conducted between October 2018 and September 2019 with face-to-face interviews all over Germany. Computer-assisted personal interviews (CAPI) and computer-assisted self-administered interview (CASI) components with a large self-administered component were used due to the sensitive topics of the survey.

The sample consisted of German speaking persons living in private households in the Federal Republic of Germany, aged between 18 and 75 years [21]. To draw a target sample size of about 5,000 respondents, which was estimated for sufficient statistical power, a potential sample of 17,186 individuals (with names and addresses) were proportionally distributed over 200 representative municipalities (sample points) in Germany. Face-to-face interviews were conducted with 4,955 respondents in these 200 representative sample points. 18 to 35-year-old persons were oversampled (n = 2,000, 40% of the total sample size) as this age group represents the part of the population sexually most active and most vulnerable to sexually transmitted infections. Thus, more detailed insights into sexual risk taking as well as sexual and reproductive health were possible for this age group. A complex sampling design was used in the GeSiD-study in order to increase the representativeness of the sample. Therefore, the dataset was weighted for age, gender, education, nationality, and region (for further details please see [21]).

All respondents gave written informed consent to take part in the study and received 30€ as a token of appreciation. The questionnaire was pretested with a sample of 1,155 respondents in 2016 and 2017 [22] and included 260 questions that focused on different aspects of sexual behavior, sexual attitudes and sexual relationships. The final sample consisted of N = 4,955 individuals.

According to the 2016 guidelines of the American Association for Public Opinion Research (AAPOR), the response rate was calculated and adapted for use in German public opinion surveys; it reached 30.2% [23, 24]. The GeSiD study protocol was approved by the Ethical Board of the Hamburg Psychotherapy Chamber (reference number: 07/2018-PTK-HH).

### Measures

Respondents were asked to indicate the extent to which they would accept seven statements on sexual behavior on a 5-point Likert scale (1 = not at all acceptable to 5 = totally acceptable). The introduction phrase was: “People may have different opinions on what is and what is not acceptable in the areas of love, sexuality and family planning.” Afterwards respondents were asked to rate the acceptability of the following statements:

- “A married person has sex with someone outside their marriage.” (extramarital sex), as an indicator for the function of marriage within a culture [25],
- “Two men have consensual sex with each other.” (consensual sex between men) respectively “Two women have consensual sex with each other.” (consensual sex between women) as an indicator for gender equality [26], and sexual comfort [27],
- “A woman has an abortion.” (abortion) and “A person has sex with a prostitute.” (sex work) as indicators for a (current) social discourse related to legal and political decisions, and
- “A person has sex with many different partners.” (promiscuity) respectively “A person has sex without love.” (sex without love) as indicators which are relevant to public health, since liberal attitudes towards casual sex may predict sexually transmitted disease risk [28].

In light of the existing literature on attitudes towards sexual behaviors, we chose age, gender, highest educational degree (sociodemographic factors) and migration background as well as religious aspects (sociocultural factors) as predictors [4, 10, 12, 15]. Gender was surveyed in two steps. First, the sex assigned at birth was asked and in a second step, the subjective gender affiliation [29]. For current analyses, educational degree was grouped into three categories of low (no school-leaving qualification or lower secondary school, still in school, no specification), middle (secondary), and high (up to university degree) education. Age was grouped from 18 to 25 and then in increments of ten years to 75 years. This resulted in six age groups allowing a differentiated analysis of linear and non-linear associations with sexual attitudes. As a sociocultural indicator, migration background was included. In Germany, migration background is defined as oneself or at least one parent not having the German citizenship at birth [30]. A distinction was made between “no migration background”, “first generation” (persons with an own migration experience), and “second generation” migrants (persons with migration background born in Germany).

Further, respondents were asked about their religious affiliation. Answering categories were “Catholic”, “Protestant”, “Muslim”, “Jewish”, “other religious affiliation (free text)” and “no religious affiliation”. Subsequently, respondents were asked how often they practice their faith with the response options ranging from 1 = never to 4 = often. Those respondents who answered that they do not belong to any religious community in the preceding question, were assigned “never” as their frequency of practicing faith in the second question.

### Statistical analyses

Descriptive analyses inform about the distribution of sample characteristics and of the responses concerning sexual attitudes for the entire sample. Binary multiple logistic regression analyses were applied to analyze variations in attitudes towards sexual behavior according to sociodemographic and sociocultural factors. For this purpose, the five-point Likert scale of the dependent variables measuring sexual attitudes were binary coded with 1 to 3 representing “rather not acceptable” (coded as 0) and 4 to 5 “rather acceptable” (coded as 1). Thereby, it is assumed that those who chose the middle category are skeptical, but do not want to state this explicitly. All independent variables were entered into the regression model simultaneously to adjust for the other covariates. The reference categories of the independent variables were defined as female gender, age 66–75 years, low education, no migration background, no religious affiliation and never practiced faith. Odds ratios, 95% confidence intervals and Nagelkerkes R^2^ were calculated. All analyses were performed using IBM SPSS Statistics (Version 27). In order to adjust for data weighting, clustering, and stratification, the module for complex samples of the IBM SPSS statistical software package was used. Concerning missing values, all available data were used (tablewise exclusion).

## Results

The distribution of the included sociodemographic and sociocultural characteristics of the sample is displayed in Table 1. As can be seen, the proportion of women and men was balanced. The sample contained slightly more persons with a high level of education compared to those with low or middle level of education (36.4%). About three-quarters said they had no migration background, 15.9% were first-generation migrants, 9.8% second-generation. A large proportion of respondents were either Protestant (27.3%) or Catholic (28.8%), 5% said they were Muslim. Of those who belong to a religious affiliation, the majority practiced faith seldom (34.5%) or sometimes (24.9%).

**Table 1:** Distribution of sociodemographic and sociocultural characteristics of the sample (weighted): German Health and Sexuality Survey (GeSiD).

	Distribution in the sample (%)
**Gender (n = 4,955)**	
Female	49.8
Male	50.2
**Age groups (n = 4,955)**	
18–25 years	12.0
26–35 years	17.6
36–45 years	16.4
46–55 years	21.8
56–65 years	18.9
66–75 years	13.3
**Education (n = 4,955)**	
Low	31.8
Middle	31.8
High	36.4
**Migration background (n = 4,926)**	
No migration background	74.3
1. generation	15.9
2. generation	9.8
**Religious affiliation (n = 4,910)**	
Protestant	27.3
Catholic	28.8
Muslim	5.3
Other religious affiliation	4.9
No religious affiliation	33.7
**Practicing faith, if religious affiliation exists (n = 3,244)**	
Never	18.7
Seldom	34.5
Sometimes	24.9
Often	21.9

Table 2 informs about the responses regarding the items measuring attitudes towards sexual behavior. Acceptance towards the topics “consensual sex between men” and “consensual sex between women” was most pronounced (category 4 or 5 on the scale). About half of the respondents accepted abortion, sex work and sex without love and 36.2% of respondents considered promiscuity acceptable. The item “extramarital sex” received the least approval with 16.0%. The proportion of missing values (“Prefer not to say”) ranged from 2.6% (promiscuity) to 5.4% (abortion).

**Table 2:** Acceptance regarding sexual behavior (weighted sample): German Health and Sexuality Survey (GeSiD).

	(1) not at all acceptable	(2)	(3)	(4)	(5) totally acceptable	Prefer not to say
Extramarital sex (n = 4,810)	2,314	837	869	374	417	145
	(46.7%)	(16.9%)	(17.5%)	(7.6%)	(8.4%)	(2.9%)
Consensual sex between men (n = 4,790)	848	273	531	431	2,708	165
	(17.1%)	(5.5%)	(10.7%)	(8.7%)	(54.6%)	(3.3%)
Abortion (n = 4,689)	700	459	1,085	709	1,736	266
	(14.1%)	(9.3%)	(21.9%)	(14.3%)	(35.0%)	(5.4%)
Sex work (n = 4,757)	876	532	1,082	804	1,464	198
	(17.7%)	(10.7%)	(21.8%)	(16.2%)	(29.5%)	(4.0%)
Consensual sex between women (n = 4,810)	503	259	586	500	2,962	145
	(10.2%)	(5.2%)	(11.8%)	(10.1%)	(59.8%)	(2.9%)
Promiscuity (n = 4,824)	1,439	678	915	653	1,139	131
	(29.0%)	(13.7%)	(18.5%)	(13.2%)	(23.0%)	(2.6%)
Sex without love (n = 4,802)	691	463	980	719	1,948	153
	(14.0%)	(9.4%)	(19.8%)	(14.5%)	(39.3%)	(3.1%)

In Table 3, dichotomized attitudes towards sexual behavior were regressed on the sociodemographic and sociocultural factors. Male and female respondents significantly differed concerning their acceptance for all sexual behaviors: While male respondents were more likely to approve extramarital sex, sex work, promiscuity and sex without love, they were less likely to accept abortion and consensual sex between two men or between two women.

**Table 3:** Associations between sociodemographic, sociocultural factors and acceptance towards sexual behavior*: Odds Ratios (OR) and 95% confidence intervals (CI) (weighted data): German Health and Sexuality Survey (GeSiD).

	Extramarital sex (n = 4,748)	Consensual sex between men (n = 4,724)	Abortion (n = 4,635)	Sex work (n = 4,696)	Consensual sex between women (n = 4,743)	Promiscuity (n = 4,762)	Sex without love (n = 4,762)
	Proportion of acceptance: 16.0%	Proportion of acceptance: 63.3%	Proportion of acceptance: 49.3%	Proportion of acceptance: 45.7%	Proportion of acceptance: 69.9%	Proportion of acceptance: 36.2%	Proportion of acceptance: 53.8%
**Gender (reference: female)**							
male	**1.39**	**0.35**	**0.79**	**1.47**	**0.63**	**1.35**	**1.35**
	**(1.16–1.65)**	**(0.29–0.42)**	**(0.69–0.90)**	**(1.27–1.71)**	**(0.52–0.76)**	**(1.16–1.56)**	**(1.16–1.56)**
**Age groups (Reference: 66–75 years)**							
18–25 years	**0.18**	**2.63**	**0.60**	**0.76**	**3.23**	**2.33**	**2.33**
	**(0.11–0.28)**	**(1.88–3.66)**	**(0.44–0.83)**	**(0.59–0.99)**	**(2.35–4.45)**	**(1.77–3.07)**	**(1.77–3.07)**
26–35 years	**0.24**	**2.13**	**0.42**	0.81	**2.43**	**2.17**	**2.17**
	**(0.16–0.36)**	**(1.57–2.88)**	**(0.32–0.55)**	(0.62–1.05)	**(1.74–3.41)**	**(1.64–2.87)**	**(1.64–2.87)**
36–45 years	**0.67**	**2.51**	**0.60**	1.13	**2.61**	**2.32**	**2.32**
	**(0.47–0.96)**	**(1.85–3.41)**	**(0.43–0.82)**	(0.86–1.47)	**(1.86–3.66)**	**(1.77–3.05)**	**(1.77–3.05)**
46–55 years	0.81	**1.94**	0.90	1.22	**1.83**	**1.85**	**1.85**
	(0.55–1.18)	**(1.46–2.57)**	(0.66–1.22)	(0.92–1.61)	**(1.34–2.51)**	**(1.38–2.49)**	**(1.38–2.49)**
56–65 years	0.90	**1.61**	0.75	0.96	**1.53**	**1.34**	**1.34**
	(0.65–1.26)	**(1.23–2.10)**	(0.57–1.00)	(0.75–1.23)	**(1.14–2.05)**	**(1.01–1.78)**	**(1.01–1.78)**
**Education (Reference: low education)**							
high	**1.87**	**3.96**	**3.28**	**1.31**	**3.23**	**2.68**	**2.68**
	**(1.38–2.53)**	**(3.16–4.96)**	**(2.67–4.03)**	**(1.08–1.59)**	**(2.58–4.03)**	**(2.21–3.24)**	**(2.21–3.24)**
middle	1.05	**1.30**	**1.48**	1.02	**1.36**	1.15	1.15
	(0.80–1.39)	**(1.06–1.59)**	**(1.22–1.81)**	(0.83–1.27)	**(1.10–1.68)**	(0.92–1.44)	(0.92–1.44)
**Migration background (Reference: no migration background)**							
2. generation	1.31	0.80	1.02	1.07	**0.73**	1.02	1.02
	(0.95–1.80)	(0.60–1.07)	(0.78–1.34)	(0.84–1.36)	**(0.55–0.97)**	(0.80–1.30)	(0.80–1.30)
1. generation	1.15	**0.26**	**0.62**	**0.67**	**0.29**	**0.65**	**0.65**
	(0.84–1.57)	**(0.20–0.34)**	**(0.49–0.78)**	**(0.54–0.84)**	**(0.22–0.37)**	**(0.53–0.80)**	**(0.53–0.80)**
**Religious affiliation (Reference: no religious affiliation)**							
Protestant	0.75	**1.41**	0.93	**1.29**	1.18	1.07	1.07
	(0.54–1.06)	**(1.02–1.95)**	(0.71–1.23)	**(1.00–1.66)**	(0.80–1.74)	(0.85–1.35)	(0.85–1.35)
Catholic	**0.66**	**1.60**	**0.64**	1.16	1.45	0.99	0.99
	**(0.47–0.94)**	**(1.14–2.23)**	**(0.49–0.83)**	(0.90–1.49)	(1.00–2.10)	(0.77–1.27)	(0.77–1.27)
Muslim	**0.35**	**0.49**	**0.33**	0.65	**0.40**	**0.43**	**0.43**
	**(0.14–0.86)**	**(0.28–0.85)**	**(0.21–0.52)**	(0.40–1.04)	**(0.23–0.68)**	**(0.25–0.74)**	**(0.25–0.74)**
Other religious affiliation	0.71	1.29	0.74	1.17	0.97	0.83	0.83
	(0.39–1.29)	(0.79–2.11)	(0,44–1,22)	(0.75–1.82)	(0.58–1.62)	(0.52–1.32)	(0.52–1.32)
**Practicing faith (Reference: no religious affiliation/never practiced faith)**							
Often	**0.60**	**0.32**	**0.29**	**0.42**	**0.32**	**0.38**	**0.38**
	**(0.41–0.89)**	**(0.23–0.43)**	**(0.22–0.40)**	**(0.32–0.54)**	**(0.22–0.45)**	**(0.28–0.51)**	**(0.28–0.51)**
Sometimes	0.96	**0.58**	**0.63**	**0.76**	**0.57**	**0.68**	**0.68**
	(0.66–1.41)	**(0.42–0.82)**	**(0.46–0.85)**	**(0.58–0.99)**	**(0.39–0.84)**	**(0.51–0.89)**	**(0.51–0.89)**
Seldom	1.01	0.74	0.89	**0.78**	0.79	0.87	0.87
	(0.70–1.45)	(0.55–1.00)	(0.69–1.14)	**(0.62–0.98)**	(0.55–1.12)	(0.70–1.09)	(0.70–1.09)
Nagelkerkes R^2^	0.098	0.260	0.182	0.064	0.221	0.147	0.147

*1–3 = rather not acceptable (0), 4–5 = rather acceptable (1), bold = sig. difference from reference (p<0.05)

Concerning age, no clear response pattern could be found, although a trend towards more acceptance in the younger age groups regarding sex between two women or between two men, promiscuity and sex without love became apparent. On the other hand, younger age groups were less likely to accept extramarital sex.

With regard to education, a clear trend was observed: while middle education was associated with slightly more acceptance towards same sex sexual activities and abortion, acceptance was clearly and significantly higher among the highly educated compared to respondents with low education for all sexual behaviors.

Compared to respondents who did not report a migration background, the first generation of migrants were significantly less likely to accept sex between two men or between two women, abortion, sex work, promiscuity and sex without love. Second generation migrants responded with a similar level of acceptance compared to persons without migration history only showing a difference for consensual sex between women.

Respondents who stated to be of Muslim faith were significantly less likely to show accepting attitudes compared to those with no religious affiliation with one exception (sex work). Interestingly, Protestants and Catholics were more likely to accept sex between two men compared to non-believers. Catholics were less likely to accept abortions, and Protestants were more likely to accept sex work compared to those with no religious affiliation. Finally, it can be seen that the more often faith was practiced, the less accepting were the attitudes towards sexual behaviors.

The proportion of explained variance (Nagelkerkes R^2^) varied between 6.4% (sex work) and 26.0% (consensual sex between two men).

## Discussion

The present study aimed at investigating public attitudes towards sexual behavior in Germany with regards to sociodemographic and sociocultural factors. Overall, attitudes were characterized by a high level of acceptance with most acceptance towards same sex sexual activities and least acceptance towards extramarital sex which is in line with results of a pilot study for this survey [14].

Nevertheless, we observed differences in sexual attitudes according to characteristics of the respondents. Female respondents expressed more favorable views on same sex sexual activities and on abortion, whereas male respondents expressed higher acceptance concerning extramarital sex, sex work, promiscuity and having sex without love. A comparison of current results with other national and international studies shows a similar picture regarding extramarital sex [12] and homosexuality [11, 31–34]. These differences may be routed in more traditional and rigid heteronormative gender role values in men [35, 36] and a general belief that it is more acceptable for men to engage in casual sexual relationships than it is for women (sexual double-standard) [12, 37, 38].

Interestingly, no clear pattern could be observed for the different sexuality-related issues regarding age, which seems somewhat counterintuitive when assuming that increasing liberality in terms of sexual attitudes may be linked to a generational effect [39]. With regard to consensual sex between two women, acceptance increased with younger age. This response behavior was also evident for promiscuity and sex without love, although the level of acceptance was consistent for the three younger age groups. The result that younger respondents were most likely to reject extramarital sex seems to be particularly interesting. Especially the young generation (generation Z) is characterized by sex unconnected to commitment [39] and current results could be an indication of a return to more traditional values [14].

In contrast to age, a clear picture emerged with regard to education which is positively associated with accepting views on sexuality-related issues. This especially holds true for the acceptance towards same sex sexual activities, abortion and for topics related to the individual´s sexual freedom (promiscuity and sex without love). Much research has been done on the acceptance of the LGBTQ community over the past years [40] and again, education was found to be an important predictor for supportive attitudes [39, 41]. One reason could be, that education has a liberalizing effect by supporting nonconformity and promoting tolerance [42]. Apart from same sex sexual activities, this may also hold true for other areas of love and sexuality: Findings from Great Britain demonstrated that higher education was associated with more pronounced acceptance towards non-exclusivity in marriage and same sex relationships [10]. Further, results from the U.S. indicated that respondents who attended college showed more accepting attitudes towards extramarital sex and same-sex sexual intercourse [39].

In terms of religion and religious devoutness, we found that especially Muslim faith and the frequency of practiced faith were associated with more rigid views on sexual behaviors. Evidence from the U.S. showed that religious beliefs, engagement, and affiliation with conservative religions (like Muslim or Catholic faith) strongly predict views on homosexuality and same-sex marriage [43, 44]. Other international studies showed that gender role traditionalism, casual sex, abortion, and extramarital sex are also viewed more conservatively depending on how religious a person is [16, 25]. Rigid views on sexuality are closely linked to the conservatism of the respective religion [45]. The fact that Muslim faith was associated with less permissive sexual attitudes may be explained by the close integration of sexuality with religious rules in Islam [46] which holds rather conservative standards on sexuality, e.g. on premarital sex, homosexuality or casual sex [47]. The Catholic Church also holds conservative moral views on sexuality-related issues [48], has fundamental homophobic roots and is against abortion [49]. Accordingly, it was not surprising that Catholic respondents were more likely to reject extramarital sex and abortions. On the other hand, a rather surprising result was that acceptance towards sex between two men was more distinct compared to those respondents who stated to belong to no religious community. In contrast, results from the U.S. showed a strong opposition against homosexuality among young Catholic people [48, 50].

This is one of the first studies examining associations between sexual attitudes and migration background as an indicator for cultural diversity. In this regard, we differentiated between first (born abroad) and second generation migrants (having at least one parent born abroad). Interestingly, the second generation of migrants responded as accepting as those respondents with no migration background in most cases, whereas the first generation of migrants responded less accepting. These findings point to aligning sexual attitudes with the current cultural context in which value concepts of persons who were born in Germany (second generation) have adapted to values of the majority (acculturation). However, current results on acculturation processes in the context of sexual attitudes are ambiguous: U.S.-American studies with immigrant students did not support the hypothesis, that the second generation of migrants resembles the behavior of non-migrants through acculturation processes [51, 52]. Contrary, in the European context, the trend towards adaptation of values and moral concepts regarding sexuality has some support from Swedish surveys on Iranian migrants [53, 54].

### Study limitations and future research

There are some limitations against which current results must be viewed. First, although a response rate of 30.2% for face-to-face interviews can be considered satisfactory [21], a selection bias due to non-response cannot be ruled out. Further, like in all surveys on sensitive topics, results should be considered in the context of social norms, which can affect the response behavior (social desirability bias). With Nagelkerkes R^2^ varying between 0.064 (sex work) and 0.260 (consensual sex between two men), the regression models have poor to medium explanatory power [55]. This underlines the fact that attitudes (also in relation to sexual behaviors) depend on a variety of factors and our study could not reflect all factors. Therefore, in future analyses, it would be interesting to take other socioeconomic factors (like income or employment) as well as political orientations into account with regard to sexual attitudes.

Further, it has to be noted that the applied concept of migration background is characterized by a simplification; migrants are a very heterogeneous group characterized by much variability in attitudes within and between ethnic groups. Respondents who did not speak German were excluded from the survey, which further limits the results of respondents with migration background. Consequently, no statements about a specific cultural background can be made. Moreover, not all current sexuality-related behaviors could be investigated in this study, e.g. increasing consumption of porn which may also shape sexual preferences and behaviors. However, the topics we dealt with are important indicators for e.g. the place of marriage in a society, gender equality, and current social discourses also in relation to political and legal decisions.

Through the dichotomization of the items concerning sexual attitudes, results of the binary logistic regression analyses are to some extent crude because representation of attitudes may be distorted, as only two poles are mapped and not the entire spectrum on the acceptance scale. However, we decided to do so for the sake of clearness and because preconditions of linear regressions were violated.

## Conclusions

Overall, our results show that sexuality-related attitudes are shaped by a variety of factors. Education, culture and religion play an important role concerning the liberality towards sexual behaviors. Results concerning education indicate that it fosters acceptance towards sexually diverse lifestyles [56]. First-generation migrants, those who belonged to the Muslim faith community, and those who frequently practice faith responded most conservatively indicating that religiosity may act as an important moral compass. Accepting response behavior of the second generation of migrants indicates acculturation processes among this subgroup with regard to sexual attitudes. Because sexual attitudes are closely linked to behavior [39], they are an important indicator for various public health outcomes such as the prevention of sexual transmitted diseases [57], mental health [58], and relationship outcomes [59]. For example, our results indicate that there is a sexual double standard for men and women concerning what is acceptable in terms of sexual permissiveness. This may have negative implications for the well-being of men and women [60] and may lead to feelings of shame and guilt in sexually active girls and women [61]. Thus, it would be desirable to overcome taboos and discuss norms and values related to sexuality in a gender-sensitive way and in the context of an increasingly diversifying society. Therefore, comprehensive sex education should be held in an intercultural and non-discriminatory manner.
